# Hybrid 2D Supramolecular Organic Frameworks (SOFs) Assembled by the Cooperative Action of Hydrogen and Halogen Bonding and π⋯π Stacking Interactions

**DOI:** 10.3390/ijms25042062

**Published:** 2024-02-08

**Authors:** Sergey V. Baykov, Artem V. Semenov, Sofia I. Presnukhina, Marina V. Tarasenko, Anton A. Shetnev, Antonio Frontera, Vadim P. Boyarskiy, Vadim Yu. Kukushkin

**Affiliations:** 1 Institute of Chemistry, Saint Petersburg State University, 7/9 Universitetskaya Nab., 199034 Saint Petersburg, Russia; s.baykov@spbu.ru (S.V.B.); a.v.semenov@spbu.ru (A.V.S.); sonya.presnuxina.98@mail.ru (S.I.P.); v.kukushkin@spbu.ru (V.Y.K.); 2 Pharmaceutical Technology Transfer Center, Ushinsky Yaroslavl State Pedagogical University, 108 Respublikanskaya St., 150000 Yaroslavl, Russia; mkarunnaya@mail.ru (M.V.T.); a.shetnev@yspu.org (A.A.S.); 3 Departament de Química, Universitat de les Illes Balears, Crta de Valldemossa km 7.5, 07122 Palma de Mallorca, Spain; toni.frontera@uib.es; 4 Institute of Chemistry and Pharmaceutical Technologies, Altai State University, 656049 Barnaul, Russia

**Keywords:** supramolecular organic framework, noncovalent interactions, oxadiazoles, DFT

## Abstract

The *cis*- and *trans*-isomers of 6-(3-(3,4-dichlorophenyl)-1,2,4-oxadiazol-5-yl)cyclohex-3-ene-1-carboxylic acid (*cis*-**A** and *trans*-**A**) were obtained by the reaction of 3,4-dichloro-*N*′-hydroxybenzimidamide and *cis*-1,2,3,6-tetrahydrophthalic anhydride. Cocrystals of *cis*-**A** with appropriate solvents (*cis*-**A**‧½(1,2-DCE), *cis*-**A**‧½(1,2-DBE), and *cis*-**A**‧½C_6_H_14_) were grown from 1,2-dichloroethane (1,2-DCE), 1,2-dibromoethane (1,2-DBE), and a *n*-hexane/CHCl_3_ mixture and then characterized by X-ray crystallography. In their structures, *cis*-**A** is self-assembled to give a hybrid 2D supramolecular organic framework (SOF) formed by the cooperative action of O–H⋯O hydrogen bonding, Cl⋯O halogen bonding, and π⋯π stacking. The self-assembled *cis*-**A** divides the space between the 2D SOF layers into infinite hollow tunnels incorporating solvent molecules. The energy contribution of each noncovalent interaction to the occurrence of the 2D SOF was verified by several theoretical approaches, including MEP and combined QTAIM and NCIplot analyses. The consideration of the theoretical data proved that hydrogen bonding (approx. −15.2 kcal/mol) is the most important interaction, followed by π⋯π stacking (approx. −11.1 kcal/mol); meanwhile, the contribution of halogen bonding (approx. −3.6 kcal/mol) is the smallest among these interactions. The structure of the isomeric compound *trans*-**A** does not exhibit a 2D SOF architecture. It is assembled by the combined action of hydrogen bonding and π⋯π stacking, without the involvement of halogen bonds. A comparison of the *cis*-**A** structures with that of *trans*-**A** indicated that halogen bonding, although it has the lowest energy in *cis*-**A**-based cocrystals, plays a significant role in the crystal design of the hybrid 2D SOF. The majority of the reported porous halogen-bonded organic frameworks were assembled via iodine and bromine-based contacts, while chlorine-based systems—which, in our case, are structure-directing—were unknown before this study.

## 1. Introduction

Metal–organic and covalent organic framework structures are currently applied in various fields [1], including heterogeneous catalysis [2,3], adsorption [4,5], the storage and separation of gases [6], pollutant capture [7], drug delivery [8], fuel cells and electrode materials [9], and optoelectronics applications [10]. Recent progress in the construction of framework architectures included the use of noncovalent interactions for the assembly of supramolecular organic frameworks (SOFs) from molecular building blocks. In the spectrum of noncovalent interactions [11,12,13], the most often used noncovalent force for SOF assembly is hydrogen bonding (HB). It is HB that, in many instances, provides the structure of hydrogen-bonded organic framework architectures [14,15], which have found application in energy storage [16,17], sensing [18,19], gas separation [20], photocatalysis [21,22] and biomedicine [23].

Halogen bonding (XB) is another noncovalent force, which was only recently applied for the crystal design of supramolecular halogen-bonded organic frameworks (XOFs) [24,25,26,27,28]. Some XOF architectures have already been successfully utilized for the adsorption of vapors of water, acetic and propionic acids [29,30], for semiconductor design [31], for iodine capture and for the detection of explosives [32]. They have also been employed as stoichiometric reagents for the conversion of arylboronic acids to the corresponding aryl iodides [33]. 

In some cases, the joint action of two different noncovalent forces leads to the assembly of hybrid SOFs [30,34,35]. Typically, in a hybrid SOF, one type of interaction has a decisive contribution to the supramolecular motif, while the others play supporting roles. A remarkable example is the 2D XOF, which was constructed by the Br⋯O XB-directed self-assembly of tetrabromobenzene-1,4-dicarboxylic acid, but its ability to capture and hold polar organic solvents was determined by the HB that occurred between the host and guest molecules [36]. In rare instances, both HB and XB equally contribute to the self-assembly of the SOF [34]; the structure-directing HB (or XB) interactions can be additionally supported by π⋯π stacking [16,37,38,39]. In the context of this study, it is noteworthy that hybrid SOFs, which are formed by the cooperative action of all three interactions (HB, XB, and π⋯π stacking), were not reported to the best of our knowledge. It is therefore clear that studies of hybrid SOFs, an understanding of the driving forces of their occurrence, and the discovery of new supramolecular synthons comprise promising goals from the materials science viewpoint. 

**Figure 1:**
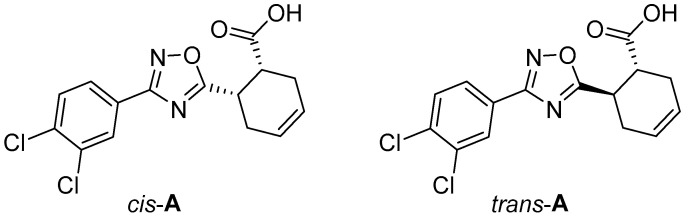
Studied compounds.

In a continuation of our work on 1,2,4-oxadiazoles [40,41] and their supramolecular [42,43] chemistry, we prepared *cis*- and *trans*-isomers of 6-(3-(3,4-dichlorophenyl)-1,2,4-oxadiazol-5-yl)cyclohex-3-ene-1-carboxylic acid (*cis*-**A** and *trans*-**A**; Figure 1) and studied their crystallization from 1,2-dichloroethane (1,2-DCE), 1,2-dibromoethane (1,2-DBE), and a *n*-hexane/CHCl_3_ mixture (1:1, *v*/*v*). 

Appropriate X-ray diffraction studies (XRD) have revealed that *cis*-**A**, on the crystallization furnished solvates *cis*-**A**‧½(1,2-DCE), *cis*-**A**‧½(1,2-DBE), and *cis*-**A**‧½C_6_H_14_, all exhibit a hybrid 2D SOF structure. This structure is built up by the cooperative action of O–H⋯O HB, Cl⋯O XB, and π⋯π stacking; the latter two types of noncovalent interactions are supportive in terms of the energy contribution (see Section 2.3 for appropriate theoretical data), but are still structure-directing forces. The self-assembled molecules of *cis*-**A** divide the space between the 2D SOF layers into infinite hollow tunnels incorporating solvent molecules. In contrast, the isomeric compound *trans*-**A** during crystallization provides single crystals, which do not exhibit a 2D SOF architecture, and the corresponding structure is built up by the combined action of HB and π⋯π stacking, without any involvement of XB. All our experiments on the design of hybrid 2D SOFs—assembled by the cooperative action of hydrogen and halogen bonding and π⋯π stacking interactions—are consistently discussed in the following sections.

## 2. Results and Discussion

### 2.1. The Hybrid 2D SOF Architectures

**Figure 2:**
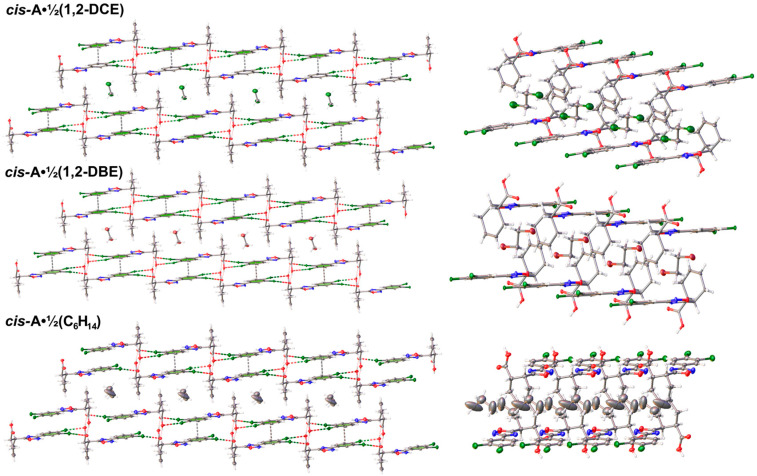
General view of the crystal structures of *cis*-**A**‧½(1,2-DCE), *cis*-**A**‧½(1,2-DBE), and *cis*-**A**‧½C_6_H_14_. Only two 2D SOF layers are shown.

The self-assembly of oxadiazole *cis*-**A** gives the solid 2D SOF architecture; the XRD structures of *cis*-**A**‧½(1,2-DCE), *cis*-**A**‧½(1,2-DBE), and *cis*-**A**‧½C_6_H_14_ are well reproduced during repeated crystallizations. In the structures, the molecules of *cis*-**A** function as tunnel walls between the 2D SOF layers, in which solvent molecules are arranged into infinite 1D chains (Figure 2); hexane in *cis*-**A**‧½C_6_H_14_ is disordered. At room temperature (or higher), all three solvates gradually lose the solvent and the crystals collapse. 

**Figure 3:**
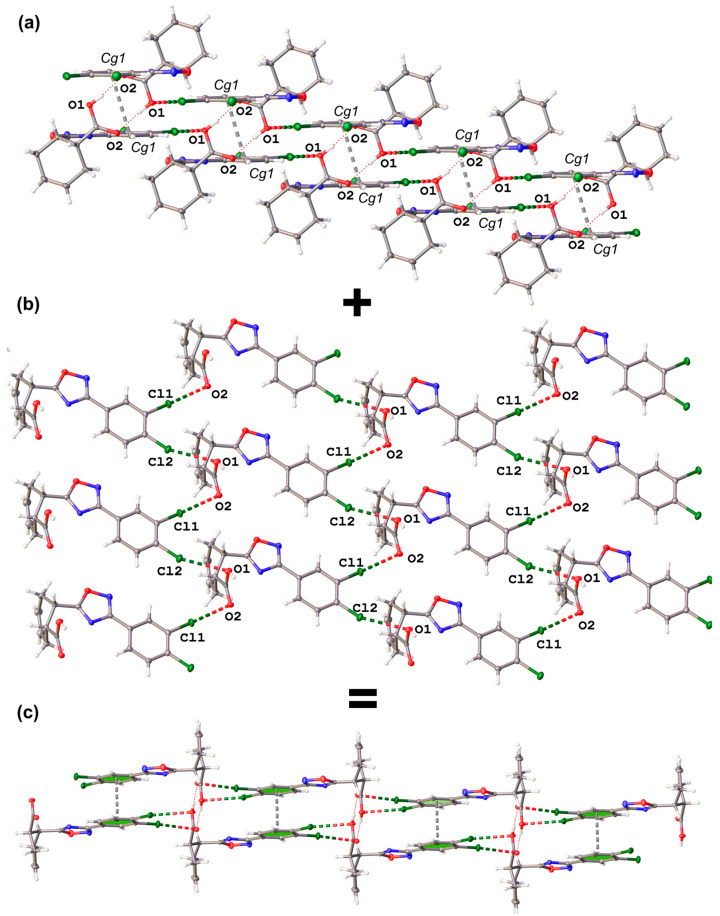
Structure-determining interactions: (**a**) O–H⋯O HB and π⋯π stacking; (**b**) Cl⋯O XB; (**c**) the resulting hybrid 2D SOF structure.

The structures of the 2D SOF do not depend on the identity of the captured solvent. All three solvates are isostructural and exhibit the same space group (*P*-1), with one molecule of *cis*-**A** per unit cell; the crystal lattice parameters of all structures are given in the ESI (Tables S1 and S2). The main noncovalent interactions in these structures are as follows: O–H⋯O HB occurred between the carboxylic groups of neighboring molecules; Cl⋯O XB occurred with both O-atoms of the carboxylic group; and π⋯π stacking occurred between the 3,4-dichlorophenyl moieties (*Cg1* planes; Figure 3). 

The Cl⋯O XB provides the 2D motif (Figure 3b), whereas HB and π⋯π stacking hold two rows of *cis*-**A,** together forming a layered 2D supramolecular motif (Figure 3a). The geometrical parameters of the corresponding noncovalent interactions are gathered in Table 1. The formulation of the structure-determining O–H⋯O HB is fully consistent with the IUPAC definition of this noncovalent interaction [44]. 

As far as XB is concerned, the geometrical parameters of all the solvates fulfill the IUPAC distance and angle criteria [46] for the identification of XB. Notably, the majority of the reported porous XB-involving organic frameworks were assembled via iodine [26,28,30,32,33,34,35,47] and bromine [31,36,48]-based XB, while chlorine-based systems—which, in our case, provide the structure of the hybrid 2D SOF structures—were unknown before this study. 

In the structures of *cis*-**A**‧½(1,2-DCE) and *cis*-**A**‧½(1,2-DBE), the interplanar distances between the two 3,4-dichlorophenyl moieties (*Cg1*⋯*Cg1*, Table 1) are equal (within the 3σ criterium) and fall in the typical range (3.41–3.61 Å) for conventional π⋯π stacking [49,50]. In the case of *cis*-**A**‧½C_6_H_14_, the *Cg1*⋯*Cg1* interplanar distance is slightly larger (3.636(2) Å) than this range. The detailed geometrical parameters of these stacking interactions are collected in Table S4 (the ESI, Section S3).

The appropriate DFT calculations that were conducted verified the availability of bond critical points for all interactions; for further details of the theoretical study, see Section 2.3.

**Figure 4:**
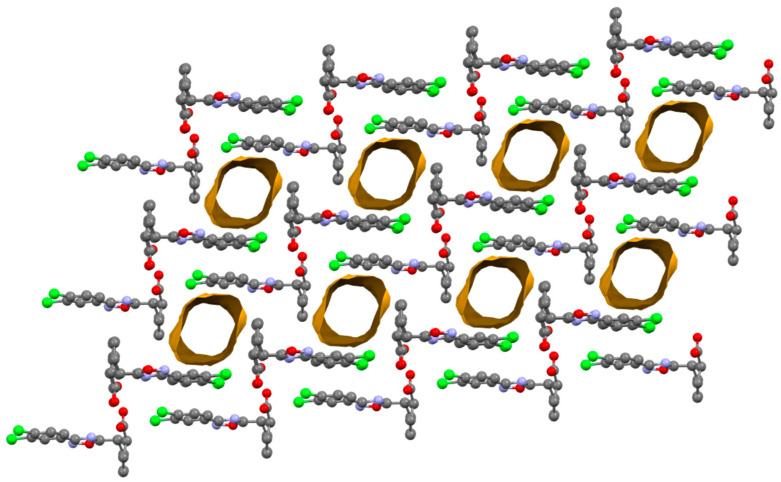

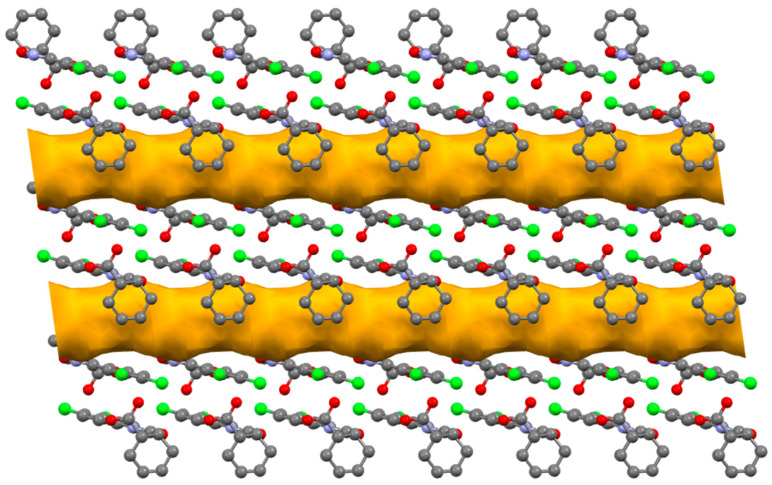
Partial representation (Mercury 4.3.1, ball and stick) of the crystal packing of *cis*-**A**‧½(1,2-DCE) after orientations evidencing the cylindrical shape and the parallel arrangement of the channels (contact surfaces in ocher). A probe radius of 1.2 Å and an approximate grid spacing of 0.7 Å were used to generate channels. Solvent molecules in the voids and H-atoms are omitted for the sake of clarity. Color coding: grey, carbon; red, oxygen; blue, nitrogen; green, chlorine.

The SQUEEZE procedure was applied to demonstrate the presence of void channels in the obtained cocrystals and to calculate their empty volume using the *cis*-**A**‧½(1,2-DCE) structure as a model (Figure 4); this view is nearly identical for all three structures and the views of the other two structures are given in Figures S1 and S2, the ESI). The channels in all cocrystals exhibit a cylindrical shape with a diameter of 8 Å; the percentage of empty volume is 14.8% in *cis*-**A**‧½(1,2-DCE) and 15.3% in both *cis*-**A**‧½(1,2-DBE) and *cis*-**A**‧½C_6_H_14_. 

### 2.2. XB-Free Structure of trans-**A**

**Figure 5:**
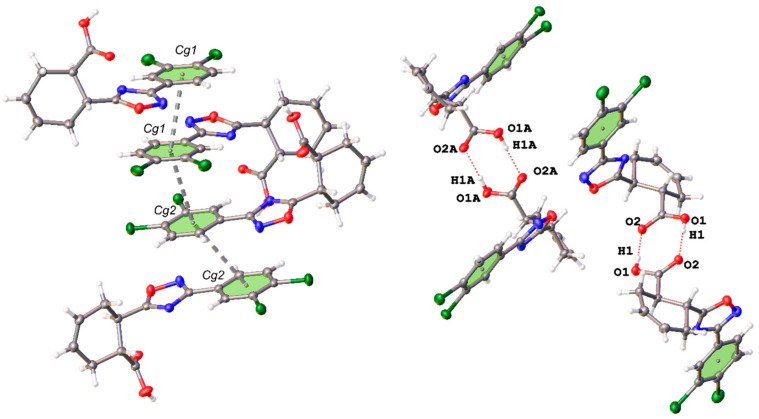
Noncovalent interactions in the structure of *trans*-**A**.

Although the main observation of this work concerns the hybrid 2D SOF (Section 2.1), for the sake of ensuring the completeness of the entire study, in this section, we briefly discuss the XRD structure of the isomeric compound *trans*-**A**. It crystallizes as a mono-component crystal with no captured solvent. Its unit cell consists of two crystallographically independent molecules exhibiting a complicated packing pattern. In the crystal structure of *trans*-**A**, we identified the conventional HB-based pairing of the carboxylic groups (for recent relevant examples, see refs. [51,52,53]) and several types of π⋯π stacking interactions between the 3,4-dichlorophenyl moieties (Figure 5; Tables S3 and S5). 

Remarkably, in contrast to the structures of *cis*-**A**, XB involving a Cl atom of the oxadiazole did not occur. Although HB is more significant than XB from an interaction energy viewpoint according to the DFT calculations (Section 2.3), the latter is important for the construction of the 2D architecture.

**Figure 6:**
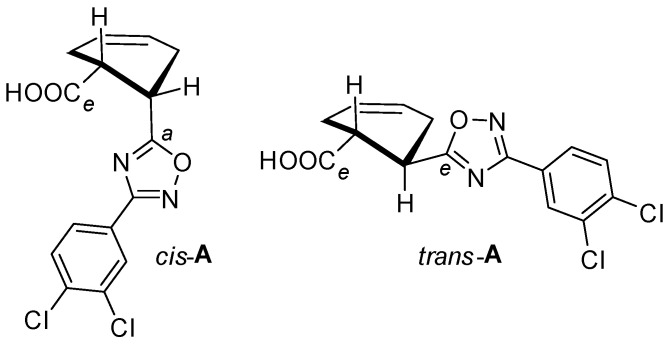
Orientation of carboxylic group and the oxadiazole core in the structures of *cis-***A** and *trans-***A**.

The comparison of the crystal structure geometry of *trans*-**A** and *cis*-**A** revealed that the carboxylic group and the oxadiazole ring are in different positions (Figure 6). In particular, in the structure of *trans*-**A,** both substituents are located in a pseudo-equatorial position. In the *cis*-isomer, by contrast, the carboxylic group is also located in a pseudo-equatorial position, but the heterocyclic ring has a pseudo-axial arrangement. As follows from the consideration of the solid architectures of *cis*-**A**, this pseudo-axial arrangement is mostly responsible for the occurrence of XB. 

### 2.3. Theoretical Considerations

To deepen our understanding of the noncovalent interactions that occur in the design of SOF architectures, we conducted theoretical calculations to estimate the contributions of different interaction energies. Analysis of the relevant literature suggests that the Cl atom is a modest σ-hole donor, particularly when compared to heavier group elements like Br and I [54]. 

**Figure 7:**
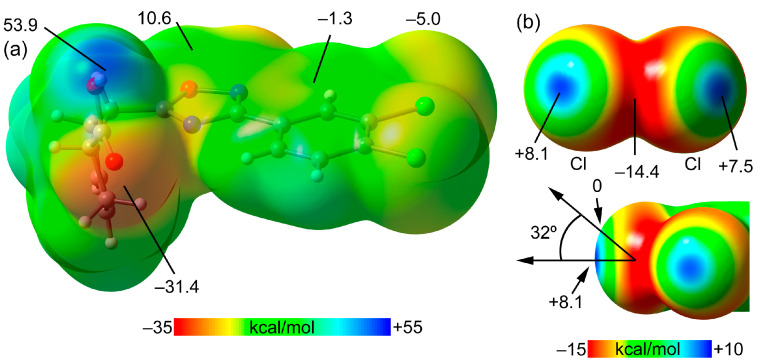
(**a**) MEP surface of *cis*-**A**; (**b**) MEP surface of *cis*-**A** focusing on the 1,2-dichlorine region with indication of the cone angle. Values are in kcal/mol.

Initially, we examined the existence and strength of σ-holes at the Cl atoms in *cis*-**A** by using molecular electrostatic potential (MEP) analysis (Figure 7). The MEP maximum, expectedly, is located at the acidic H-atom of the carboxylic group (+53.9 kcal/mol), while the minimum is at the O-atom of the same group (–31.4 kcal/mol), indicating a likelihood of energetically favorable OH⋯O HB. The MEP over the six-membered aromatic ring is low (−1.3 kcal/mol), favoring π-stacking interactions due to the minimal electrostatic repulsion. Moreover, positive MEP values at the center of the oxadiazole ring indicate a preference for antiparallel π-stacking via dipole–dipole attraction. We further explored σ-holes at the Cl atoms by focusing on the MEP surface of the dichlorobenzene fragment at a reduced scale (Figure 7). Here, the MEP values are modest (+8.1 and +7.5 kcal/mol), as expected for the Cl atom, with negative belts around −5.0 kcal/mol perpendicular to and −14.4 kcal/mol in the molecular plane. The σ-hole cone angle is 32°, suggesting that electron-rich atoms must approach the chlorine at an angle between 148 and 180° for effective interaction, as is the case for the ∠C–Cl⋯O angles (Table 1).

**Figure 8:**
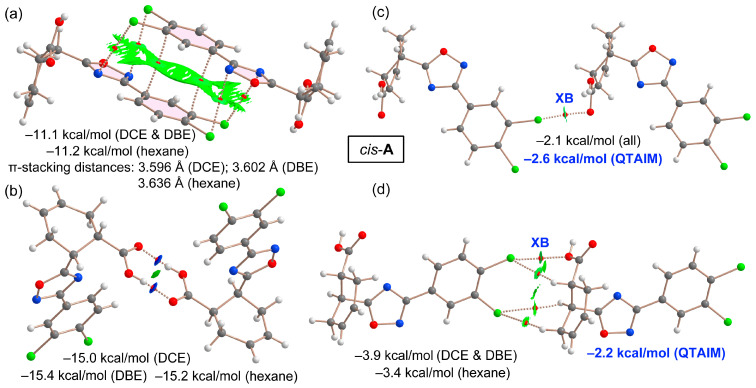
QTAIM (BCP in red and bond paths as dashed bonds) and NCIPlot (RDG = 0.5, ρ cut-off = 0.04, color scale −0.035 (blue) ≤ (signλ_2_)ρ ≤ 0.035 (red) for the π-stacked (**a**) HB (**b**) and XB (**c**,**d**) dimers. Only intermolecular interactions are represented. The π-stacking distances are indicated and measured using the ring centroids.

Our comprehensive QTAIM and NCIplot analysis of four dimers of *cis*-**A**, as identified in the XRD structures of their solvates, aimed to assess the relative strength of HB, XB, and π-stacking in the solid state. As Figure 8 illustrates, despite the presence of various solvents, the interaction energies for these dimers remain consistent, indicating that solvent molecules do not significantly impact the strength of these interactions. The π⋯π dimer analysis (Figure 8a) reveals two bond critical points (BCP), bond paths, and a broad green RDG (reduced density gradient) isosurface, characteristic of π-stacking (see theoretical methods for the terminology used herein regarding bond paths and critical points). Additionally, two BCP and bond paths connect the Cl atoms of one molecule to the five-membered ring of the other (and vice versa), facilitated by the electrostatic attraction between the π-acidic oxadiazole and the Cl atoms’ negative belts, as corroborated by the MEP analysis. The interaction energy for this π-stacking dimer ranges from −11.1 to −11.2 kcal/mol. For the H-bonded dimer (Figure 8b), which forms the *R*_2_^2^(8) motif, two symmetric BCPs and bond paths are observed, as well as a dark blue RDG isosurface for each H-bond. The dark blue color is an indication of a strong HB. This agrees with the computed interaction energies, which are between −15.0 and −15.4 kcal/mol, consistent with the previous MEP results. 

The first XB analysis, focusing on the Cl1⋯O2 interaction (Figure 8c), reveals a single BCP, bond path, and green RDG isosurface for this dimer, with a modest interaction energy of −2.1 kcal/mol across all solvates. This aligns with the small MEP value at the chlorine’s σ-hole. Utilizing the QTAIM method proposed by Bartashevich and Tsirelson [55], the XB energy is estimated at −2.6 kcal/mol, supporting these findings. Lastly, the dimer with the Cl2⋯O1 interaction (Figure 8d) exhibits, besides XB, three CH⋯Cl contacts, each characterized by BCP, bond paths, and small green RDG isosurfaces. The total interaction energy for this dimer ranges from −3.4 to −3.9 kcal/mol, encompassing both the XB and HB. The QTAIM-estimated XB contribution is −2.2 kcal/mol, which is slightly weaker than the Cl1⋯O2 contact; this is consistent with its longer distance and smaller angle, as detailed in Table 1 (Section 2.1).

The DFT analysis results underscore that HB is the predominant interaction in terms of energetic significance, followed by π-stacking. The contribution of both XB interactions is approximately −4.8 kcal/mol, as deduced from QTAIM calculations, marking them as considerably weaker in comparison. Nevertheless, the Cl⋯O interactions, despite their relatively lower energy contribution, play a meaningful role in the formation of the SOF discussed in this study, as depicted in Figure 3b and this energetic analysis.

## 3. Material and Methods

### 3.1. Materials and Instruments

3,4-Dichloro-*N′*-hydroxybenzimidamide was prepared from the corresponding nitrile according to the reported procedure [56]. All other reagents and solvents were purchased and were used as received in BLDPharm (Shanghai, China), Macklin (Shanghai, China). NMR spectra were recorded on Bruker Avance DPX 400 (400 MHz and 101 MHz for ^1^H and ^13^C; Bruker Corporation, Billerica, MA, USA) in CDCl_3_. Chemical shifts are reported as parts per million (*δ*, ppm). The ^1^H and ^13^C spectra were calibrated using the residual signals of CHCl_3_ as an internal reference (7.26 and 77.16 ppm for ^1^H and ^13^C, respectively). Multiplicities are abbreviated as follows: s = singlet, d = doublet, t = triplet, q = quartet, m = multiplet, br = broad; coupling constants, *J*, are reported in Hertz (Hz). Melting points were determined in open capillary tubes on an Electrothermal IA 9300 series Digital Melting Point Apparatus (Electrothermal, Rochford, Essex, UK). The high-resolution mass spectra (HRMS) were measured on Bruker Maxis HRMS-ESI-qTOF (ESI Ionization; Bruker Corporation, Billerica, MA, USA).

### 3.2. Synthetic Procedures

***cis*-6-(3-(3,4-Dichlorophenyl)-1,2,4-oxadiazol-5-yl)cyclohex-3-ene-1-carboxylic acid** (***cis*-A**). This compound was prepared using the known protocol [57]: *cis*-1,2,3,6-tetrahydrophthalic anhydride (152 mg, 1 mmol) was added to a mixture of 3,4-dichloro-*N′*-hydroxybenzimidamide (205 mg, 1 mmol) and 1,4-dioxane (10 mL). The reaction mixture was stirred at RT for 2 h and K_2_CO_3_ (276 mg, 2 mmol) was added in one portion. The reaction mixture was then heated and stirred overnight at 100 °C, cooled to RT, and diluted with water (50 mL); this was followed by the addition of hydrochloric acid to pH ~1. The released precipitate was filtered off, washed with water (25 mL) and dried in air at RT to give *cis*-**A** in 40% yield (136 mg) as a colorless powder; mp 127–129 °C. ^1^H NMR (400 MHz, CDCl_3_) *δ* 8.15 (d, *J* = 2.0 Hz, 1H), 7.89 (dd, *J* = 8.4, 2.0 Hz, 1H), 7.55 (d, *J* = 8.4 Hz, 1H), 5.84–5.71 (m, 2H), 3.77 (td, *J* = 5.9, 3.4 Hz, 1H), 3.33 (td, *J* = 7.0, 3.5 Hz, 1H), 2.90–2.79 (m, 1H), 2.77–2.65 (m, 2H), 2.53 (dd, *J* = 17.0, 6.4 Hz, 1H). ^13^C NMR (101 MHz, CDCl_3_) *δ* 180.6, 178.4, 166.5, 135.4, 133.2, 130.9, 129.4, 126.8, 126.5, 125.4, 124.2, 40.3, 33.2, 26.8, 25.1. HRMS (ESI^+^), *m*/*z*: [M + Na]^+^ calcd. for C_15_H_12_Cl_2_N_2_O_3_Na^+^ 361.0117; found 361.0137.

***trans*-6-(3-(3,4-Dichlorophenyl)-1,2,4-oxadiazol-5-yl)cyclohex-3-ene-1-carboxylic acid** (***trans***-**A**). This compound was prepared using our previously developed method [58]: *cis*-1,2,3,6-tetrahydrophthalic anhydride (152 mg, 1 mmol) was added to a solution of 3,4-dichloro-*N*′-hydroxybenzimidamide (205 mg, 1 mmol) in DMSO (2 mL). The reaction mixture was then stirred at RT for 18 h, whereupon finely ground NaOH (80 mg, 2 mmol) was added in one portion. The reaction mixture was stirred at RT for 4 h and diluted with water (30 mL); this was followed by the addition of hydrochloric acid to pH ~1. The released precipitate was filtered off, washed with water (25 mL) and dried in air at RT to give *trans*-**A** in 68% yield (232 mg) as a colorless powder; mp 151–153 °C. ^1^H NMR (400 MHz, CDCl_3_) *δ* (d, *J* = 1.6 Hz, 1H), 7.88 (dd, *J* = 8.4, 2.0 Hz, 1H), 7.54 (d, *J* = 8.4 Hz, 1H), 5.82–5.71 (m, 2H), 3.57 (td, *J* = 10.6, 9.8, 5.8 Hz, 1H), 3.22 (td, *J* = 10.7, 9.7, 5.8 Hz, 1H), 2.68–2.51 (m, 2H), 2.48–2.32 (m, 2H). ^13^C NMR (101 MHz, CDCl_3_) *δ* 181.9, 179.6, 166.7, 135.6, 133.4, 131.1, 129.5, 126.8, 126.6, 125.1, 124.4, 41.9, 34.0, 28.9, 27.7. HRMS (ESI^+^), *m*/*z*: [M + Na]^+^ calcd. for C_15_H_12_Cl_2_N_2_O_3_Na^+^ 361.0117; found 361.0141.

### 3.3. Crystal Growth and the XRD Studies 

Cocrystals *cis*-**A**‧½(1,2-DCE), *cis*-**A**‧½(1,2-DBE), and *cis*-**A**‧½C_6_H_14_ were obtained via the slow evaporation of the corresponding solutions of *cis*-**A** in 1,2-dichloroethane, 1,2-dibromoethane, and a *n*-hexane/CHCl_3_ (1:1, *v*/*v*) mixture in air at RT. Single crystals of *trans*-**A** were grown via the slow evaporation of its 1,2-dichloroethane solution in air at RT. The XRD data for *cis*-**A**‧½(1,2-DBE), *cis*-**A**‧½C_6_H_14_, and *trans*-**A** were collected using a Rigaku SuperNova diffractometer and CuKα (λ = 0.154184 nm) radiation, whereas *cis*-**A**‧½(1,2-DCE) was studied using a Xcalibur Eos diffractometer and MoKα (λ = 0.71073 nm) radiation. The structure was solved with the ShelXT [59] structure solution program using Intrinsic Phasing and refined with the ShelXL [60] refinement program incorporated into the OLEX2 program package [61] by means of Least Squares minimization. Supplementary crystallographic data for this paper have been deposited at Cambridge Crystallographic Data Centre and can be obtained free of charge via www.ccdc.cam.ac.uk/data_request/cif (accessed on 8 January 2024) (CCDC numbers 2314867 (*trans*-**A**), 2314868 (*cis*-**A**‧½(1,2-DCE)), 2314869 (*cis*-**A**‧½(1,2-DBE)), 2314870 (*cis*-**A**‧½C_6_H_14_).

### 3.4. Computational Details

The calculation of the non-covalent interactions was carried out using the Gaussian-16 program [62] and the PBE0-D3/def2-TZVP level of theory [63,64,65]. To evaluate the interactions in the solid state, the crystallographic coordinates were used because we were interested in the evaluation of the contacts as they stand in the solid state. Therefore, single-point energy calculations were carried out. The Bader’s “Atoms in molecules” theory (QTAIM) [66,67] and noncovalent interaction plot (NCIPlot) [68] were to study the interactions discussed herein by means of the AIMAll calculation package [69]. The molecular electrostatic potential surfaces (isosurface 0.001 a.u.) were computed using the Gaussian-16 (Revision C.01) software [62]. The halogen bonding distribution was estimated using the potential energy density (V_g_) at the bond critical point and the equation proposed in the literature [55]. Given that the QTAIM calculations were conducted via single-point analyses, it should be mentioned that the Bond Critical Points (BCPs) addressed in this discussion are essentially mathematical Critical Points. Accordingly, the term “bond paths” is more accurately described as Atomic Interaction Lines (AILs). However, we opted for the more universally recognized terminology of BCPs and bond paths for ease of understanding. The final virial ratios of the wavefunctions for the 12 dimers analyzed were closely compared against the theoretical value of 2.0. Remarkably, these ratios ranged from 2.00158 to 2.00162, demonstrating an excellent alignment with the theoretical benchmark. This close correspondence to the expected value lends substantial credibility to the wavefunctions employed in the QTAIM calculations.

## 4. Conclusions

We obtained three hybrid 2D SOFs assembled by the collective action of O–H⋯O HB, Cl⋯O XB, and π⋯π stacking interactions; this is the first case of 2D SOFs that include chlorine-based XB. The DFT analysis highlights that HB is the most significant interaction, followed by π-stacking; meanwhile, the energy contribution of XB is smaller. However, XB plays the structure-directing role in the construction of 2D SOFs, as demonstrated by the comparison of the structures of *cis*- and *trans*-**A**. The comparison revealed the different orientation of the carboxylic groups and the oxadiazole rings relative to the cyclohexene moiety, and we assume that this distinction is mostly responsible for the occurrence of XB in the *cis*-**A** structures. The obtained data help enhance the cognition of the cooperation of diverse noncovalent forces (i.e., HB, HaB, and π⋯π stacking) in the self-assembly of hybrid SOFs and provide new opportunities for the targeted crystal design of such systems. The achieved results also demonstrate that polyfunctional heterocycles can be applied as useful supramolecular synthons for crystal engineering. Further research in this field could focus on the crystal design of hybrid SOFs exhibiting larger pores that are suitable for gas adsorption.

## Figures and Tables

**Table 1:** Geometrical parameters of the noncovalent interactions in the structures of *cis*-**A**‧½(1,2-DCE), *cis*-**A**‧½(1,2-DBE), and *cis*-**A**‧½C_6_H_14_.

Structure	Contact (Y–X⋯O)	d(Y–X⋯O), Å	∠(Y–X⋯O), °	R ^a^
Hydrogen bonds				
*cis*-A‧½(1,2-DCE)	O1–H1⋯O2	1.7796(13) (2.6160(16)) ^b^	173.56(9)	0.65
*cis*-A‧½(1,2-DBE)	O1–H1⋯O2	1.784(2) (2.620(2)) ^b^	173.84(13)	0.66
*cis*-A‧½C_6_H_14_	O1–H1⋯O2	1.7788 (2.616(2)) ^b^	172.81(14)	0.65
*cis*-A‧½(1,2-DCE)	O1–H1⋯O2	1.7796(13) (2.6160(16)) ^b^	173.56(9)	0.65
Halogen bonds				
*cis*-A‧½(1,2-DCE)	C12–Cl1⋯O2	3.0408(12)	175.79(6)	0.93
	C13–Cl2⋯O1	3.1820(13)	157.27(6)	0.97
*cis*-A‧½(1,2-DBE)	C12–Cl1⋯O2	3.0443(18)	173.85(9)	0.93
	C13–Cl2⋯O1	3.1701(19)	157.39(10)	0.97
*cis*-A‧½C_6_H_14_	C12–Cl1⋯O2	3.0769(18)	172.29(10)	0.94
	C13–Cl2⋯O1	3.2172(19)	156.55(10)	0.98
π⋯π Stacking				
*cis*-A‧½(1,2-DCE)	*Cg1*⋯*Cg1*^c^	3.5964(13)		
*cis*-A‧½(1,2-DBE)	*Cg1*⋯*Cg1*^c^	3.6015(19)		
*cis*-A‧½C_6_H_14_	*Cg1*⋯*Cg1*^c^	3.636(2)		

^a^ R is interatomic distance to Bondi Σ_vdW_ ratio [45], Σ_vdW_ H + O = 2.72 Å, Σ_vdW_ Cl + O = 3.27 Å; ^b^ the Y⋯O distance (Å); ^c^ *Cg1* is a plane of the 3,4-dichlorophenyl moiety.

## Data Availability

Data are contained within the article and Supplementary Materials.

## Supplementary Materials

The following supporting information can be downloaded at: https://www.mdpi.com/article/10.3390/ijms25042062/s1. Copies of ^1^H and ^13^C NMR spectra for *cis*-**A** and *trans*-**A**; X-ray diffraction data; geometrical parameters of noncovalent interactions in the structure of *trans*-**A** and voids visualizations for *cis*-**A**‧½(1,2-DBE) and *cis*-**A**‧½C_6_H_14_.
