# Supplementary material for: Hybrid 2D Supramolecular Organic Frameworks (SOFs) Assembled by the Cooperative Action of Hydrogen and Halogen Bonding and π⋯π Stacking Interactions
Source: Int J Mol Sci. 2024 Feb 8;25(4):2062. doi: 10.3390/ijms25042062 (PMC10889172; doi:10.3390/ijms25042062)
Supplement: Supplementary file 1 [file ijms-25-02062-s001.zip › ijms-2840850-supplementary.pdf]

Electronic Supporting Information

Hybrid 2D Supramolecular Organic Frameworks (SOFs) Assembled by the Cooperative Action of Hydrogen and Halogen Bonding and π···π Stacking Interactions

Sergey V. Baykov,[a] Artem V. Semenov,[a] Sofia I. Presnukhina,[a] Marina V. Tarasenko,[b] Anton A. Shetnev,[b] Antonio Frontera,[c] Vadim P. Boyarskiy,[a,*] Vadim Yu. Kukushkin[a,d]

[a]*Institute of Chemistry, Saint Petersburg State University, 7/9 Universitetskaya Nab., 199034 Saint Petersburg, Russian Federation*

[b]*Pharmaceutical Technology Transfer Center, Ushinsky Yaroslavl State Pedagogical University, 108 Respublikanskaya St., 150000 Yaroslavl, Russian Federation*

[c]*Departament de Química, Universitat de les Illes Balears, Crta de Valldemossa km 7.5, 07122 Palma de Mallorca (Baleares), Spain*

[d]*Institute of Chemistry and Pharmaceutical Technologies, Altai State University, 656049 Barnaul, Russian Federation*

Table of Contents

S1. X-ray diffraction data 3
S2. Copies of the NMR spectra of *cis*-**A** and *trans*-**A** 5
S3. Noncovalent interactions in the structure of *trans*-**A** 7
S4. Void channels in structures of *cis*-**A**·½(1,2-DBE) and *cis*-**A**·½C_6H_{14} 8

S1. X-ray diffraction data

Table S1. Crystal data and structure refinement parameters for cocrystals *cis*-**A**·½(1,2-DCE) and *cis*-**A**·½(1,2-DBE).

Structure	*cis*-**A**·½(1,2-DCE)	*cis*-**A**·½(1,2-DBE)
Identification code	BSC-231	BSC-240
CCDC number	2314868	2314869
Empirical formula	$C_{16}H_{14}Cl_3N_2O_3$	$C_{16}H_{14}BrCl_2N_2O_3$
Formula weight	388.64	433.10
Temperature, K	100(2)	100(2)
Crystal system	triclinic	triclinic
Space group	P-1	P-1
a, Å	6.9888(3)	7.0698(5)
b, Å	11.6332(6)	11.6573(7)
c, Å	12.0134(6)	11.9914(7)
α, °	66.054(5)	65.626(5)
β, °	75.188(4)	75.012(5)
γ, °	76.989(4)	76.789(5)
Volume, $Å^3$	854.80(8)	861.19(10)
Z	2	2
ρ_{calc}g, cm^3	1.510	1.670
μ, mm^{-1}	0.553	6.267
F(000)	398.0	434.0
Crystal size, mm^3	0.22 × 0.17 × 0.15	0.21 × 0.18 × 0.16
Radiation	Mo Kα (λ = 0.71073)	Cu Kα (λ = 1.54184)
2Θ range for data collection, °	6.088 to 64.674	8.238 to 152.13
Index ranges	$-10 \le h \le 10$, $-17 \le k \le 17$, $-17 \le l \le 17$	$-8 \le h \le 6$, $-14 \le k \le 14$, $-14 \le l \le 14$
Reflections collected	18152	6270
Independent reflections	5484 [R_{int} = 0.0269, R_{sigma} = 0.0316]	3492 [R_{int} = 0.0329, R_{sigma} = 0.0318]
Data/restraints/parameters	5484/0/218	3492/0/218
Goodness–of–fit on F^2	1.048	1.049
Final R indexes [I>=2σ (I)]	R_1 = 0.0446, wR_2 = 0.1130	R_1 = 0.0436, wR_2 = 0.1202
Final R indexes [all data]	R_1 = 0.0536, wR_2 = 0.1181	R_1 = 0.0455, wR_2 = 0.1222
Largest diff. peak/hole/ $eÅ^{-3}$	0.53/–0.99	1.04/–1.01

Table S2. Crystal data and structure refinement parameters for cocrystal *cis*-**A**·½C_6H_{14} and compound *trans*-**A**.

Structure	*cis*-**A**·½C_6H_{14}	*trans*-**A**
Identification code	BSC-242	BSC-221
CCDC number	2314870	2314867
Empirical formula	$C_{18}H_{17}Cl_2N_2O_3$	$C_{15}H_{12}Cl_2N_2O_3$
Formula weight	380.23	339.17
Temperature, K	100(2)	100(2)
Crystal system	triclinic	triclinic
Space group	P-1	P-1
a, Å	7.0450(3)	10.2007(3)
b, Å	11.5316(3)	11.1248(3)
c, Å	12.0752(4)	14.0504(4)
α, °	67.014(3)	73.976(2)
β, °	74.958(4)	76.447(2)
γ, °	76.945(3)	77.314(2)
Volume, $Å^3$	863.56(6)	1469.21(8)
Z	2	4
ρ_{calc}g, cm^3	1.462	1.533
μ, mm^{-1}	3.559	4.110
F(000)	394.0	696.0
Crystal size, mm^3	0.19 × 0.18 × 0.15	0.12 × 0.11 × 0.1
Radiation	Cu Kα (λ = 1.54184)	CuKα (λ = 1.54184)
2Θ range for data collection, °	8.412 to 141.232	6.66 to 142.128
Index ranges	$-8 \le h \le 8$, $-11 \le k \le 14$, $-14 \le l \le 14$	$-11 \le h \le 12$, $-12 \le k \le 13$, $-17 \le l \le 17$
Reflections collected	9185	15780
Independent reflections	3278 [R_{int} = 0.0340, R_{sigma} = 0.0379]	5601 [R_{int} = 0.0390, R_{sigma} = 0.0459]
Data/restraints/parameters	3278/0/227	5601/0/399
Goodness–of–fit on F^2	1.058	1.071
Final R indexes [I>=2σ (I)]	R_1 = 0.0496, wR_2 = 0.1359	R_1 = 0.0400, wR_2 = 0.1019
Final R indexes [all data]	R_1 = 0.0556, wR_2 = 0.1415	R_1 = 0.0469, wR_2 = 0.1065
Largest diff. peak/hole/ $eÅ^{-3}$	0.61/–0.58	0.90/–0.39

S2. Copies of the NMR spectra of *cis*-**A** and *trans*-**A**

^{1}H and ^{13}C spectra of *cis*-**A**

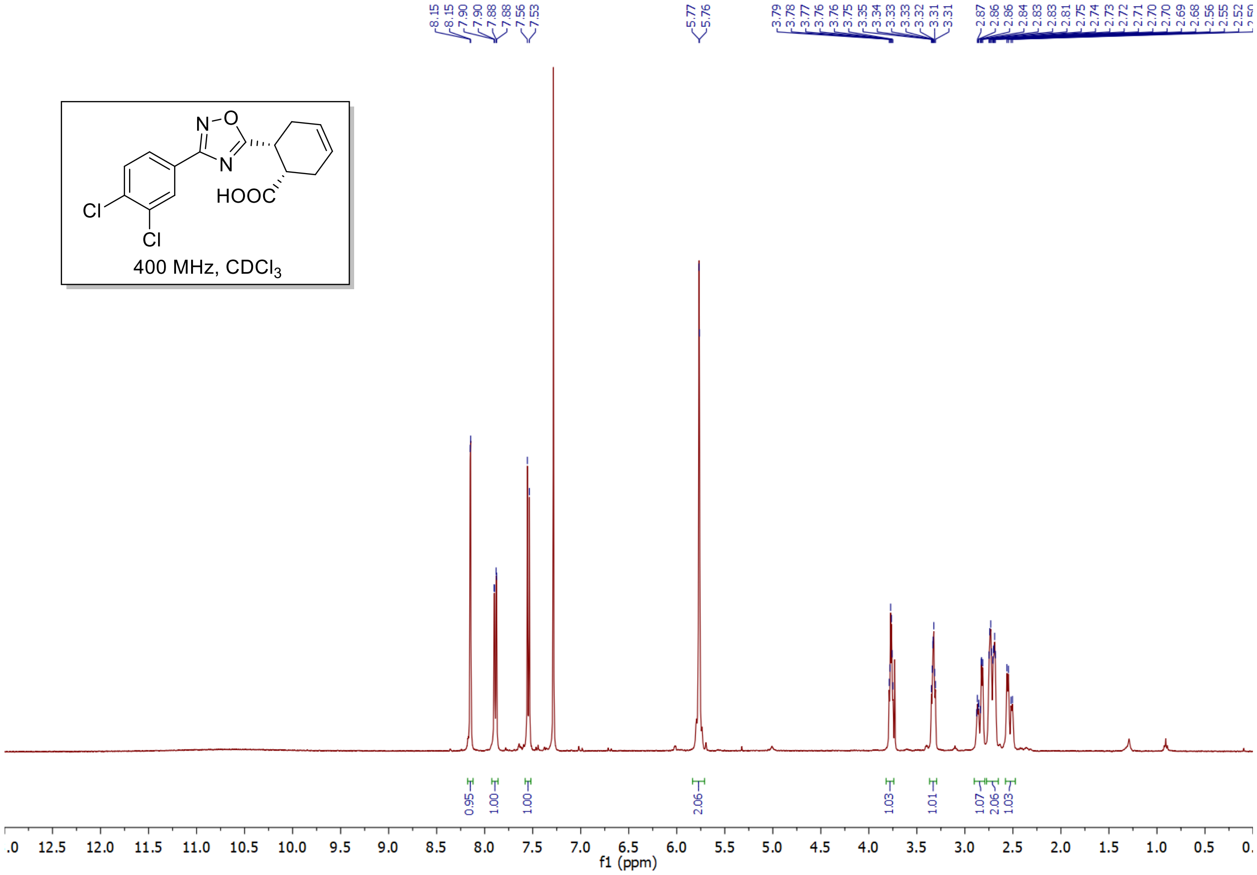

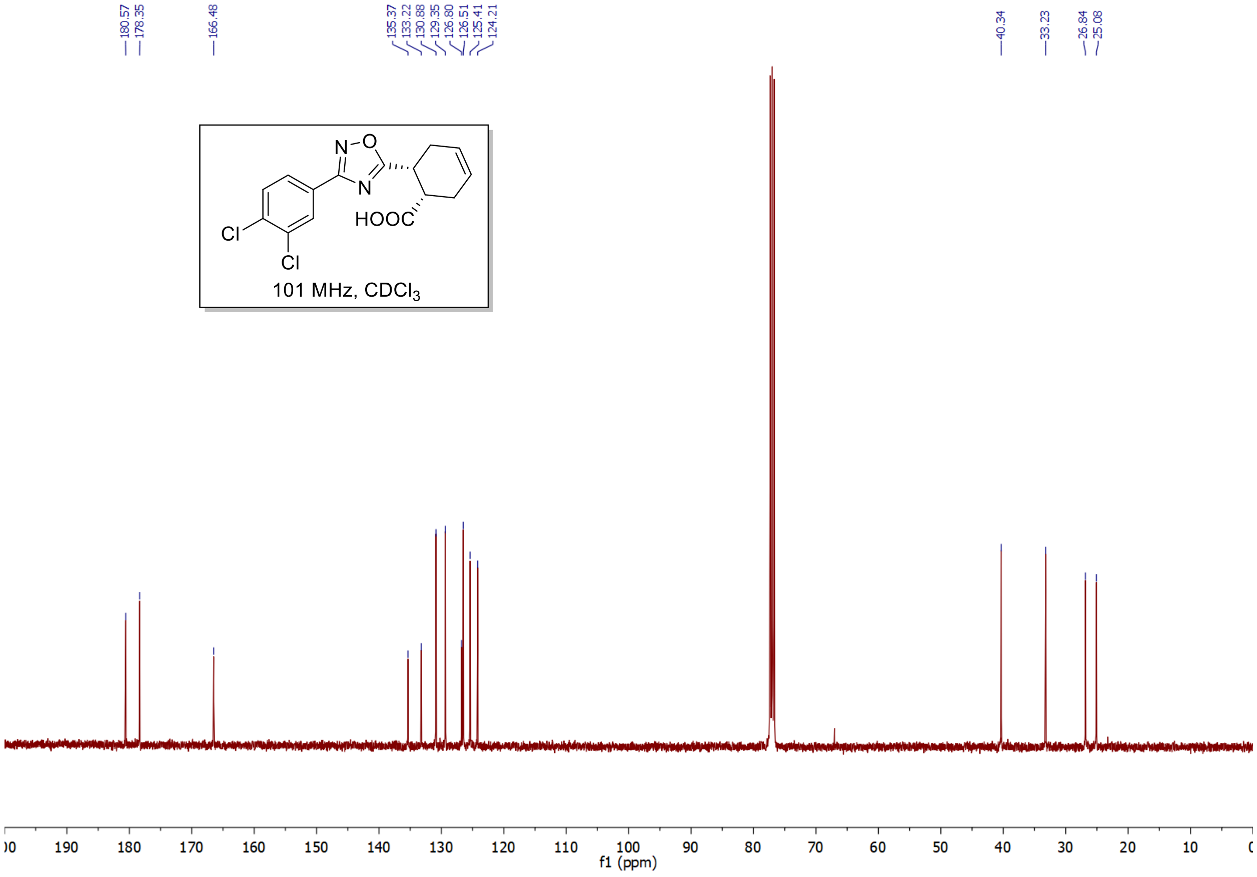

^{1}H and ^{13}C spectra of *trans*-**A**

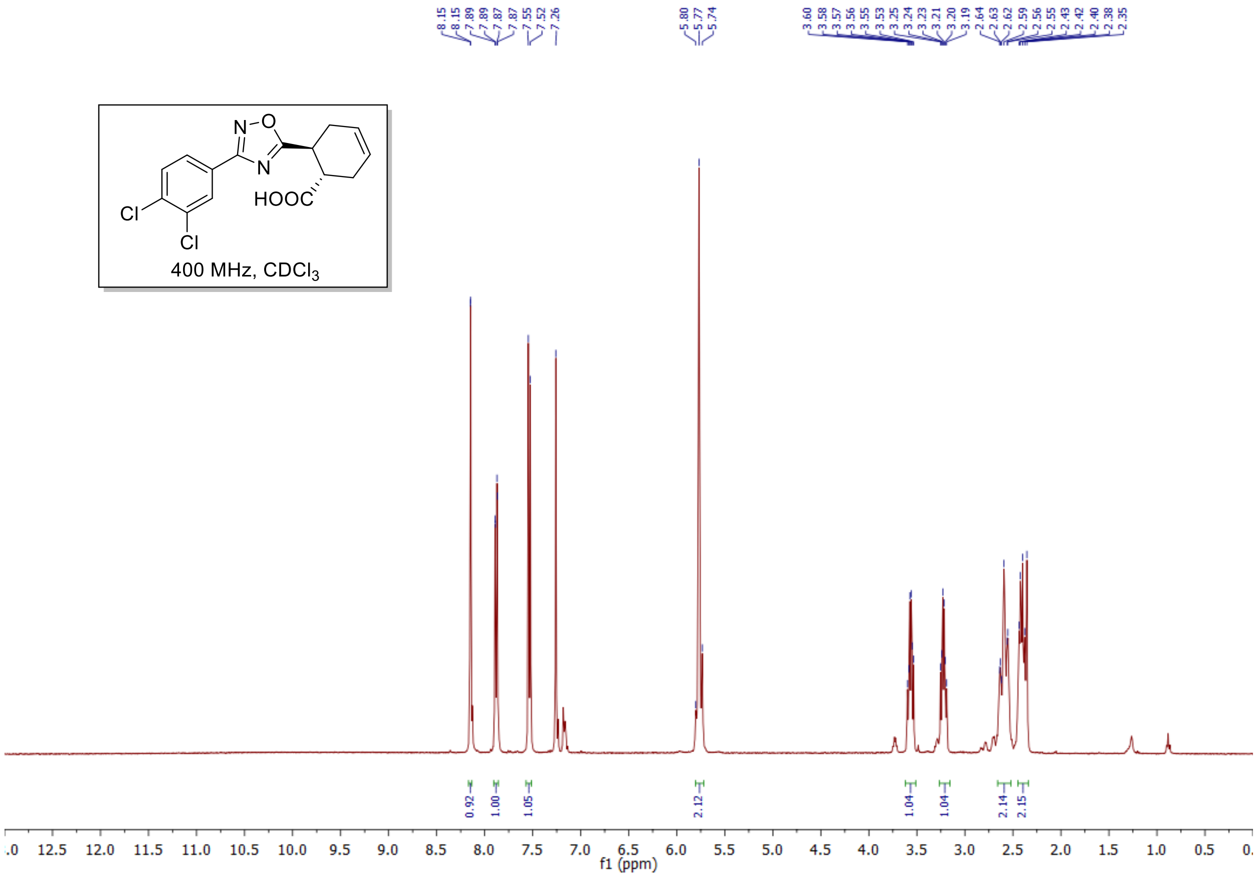

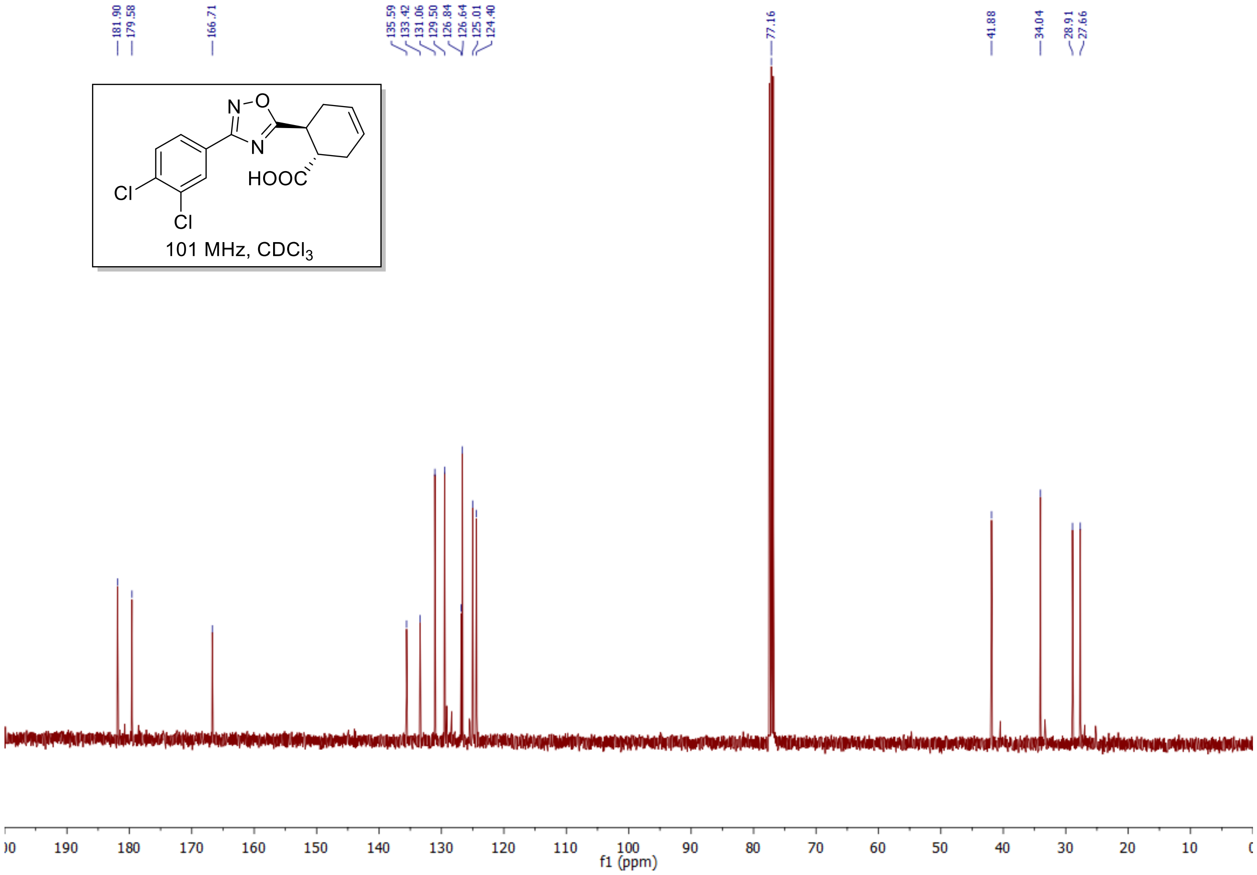

S3. Noncovalent interactions in the structure of *trans*-**A**

Table S3. Geometrical parameters of hydrogen bonds in the structure of *trans*-**A**.

**Contact**	**d(H···O), Å**	**d(O···O), Å**	**∠(O–H···O), °**	**R**[a]
O1–H1···O2	1.8123(13)	2.6512(18)	176.51(11)	0.67
O1A–H1A···O2A	1.8174(15)	2.656(2)	176.73(13)	0.67

[a] R is interatomic distance to Bondi (*J. Phys. Chem., 1964, 68, 441–451*) Σ_{vdW} ratio, Σ_{vdW} H + O = 2.72 Å.

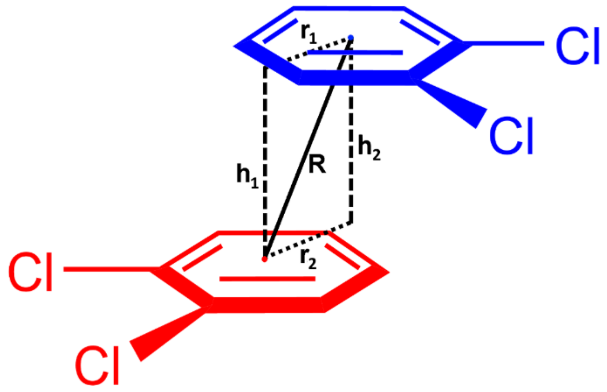

Table S4. Geometrical parameters of π···π stacking interactions in the structures of *cis*-**A**·½(1,2-DCE), *cis*-**A**·½(1,2-DBE), and *cis*-**A**·½C_6H_{14}.

Structure	**R, Å**[a]	**h, Å**[b]	**r, Å**[c]	**φ, °** [d]	**θ, °** [e]
cis-**A**·½(1,2-DCE)	3.5964(13)	3.4358(15)	1.063(3)	0.000(7)	0.00(1)
cis-**A**·½(1,2-DBE)	3.602(4)	3.394(4)	1.204(7)	0.00(2)	0.000(18)
cis-**A**·½C6H14	3.6364(16)	3.4134(18	1.254(3)	0.0(1)	0.00(7)

[a] R, distance between the centroid of one ring and the centroid of a π···π stacked ring;
[b] h_1 and h_2, distance between the centroid of one ring and the plane of a π···π stacked ring;
[c] r_1 and r_2, distance between the centroid of one ring and the projection of the centroid of a π···π stacked ring to the first plane (rings offset);
[d] φ, twist angle is defined as the angle between the plane of the anchoring ring and the plane containing six atoms of aromatic ring;
[e] θ, angle between planes of adjacent π···π stacked ring.

Table S5. Geometrical parameters of π···π stacking interactions in the structure of *trans*-**A**.

**Plane**[a]	**R, Å**[b]	**h_1, Å**[c]	**h_2, Å**[c]	**r_1, Å**[d]	**r_2, Å**[d]	**φ, °** [e]	**θ, °** [f]
Cg[f]*1···Cg1*	3.6239(17)	3.365(2)	-3.365(2)	1.345(3)	1.345(3)	0.0(3)	0.00
Cg2···Cg2	3.9102(16)	3.468(2)	-3.468(2)	1.807(3)	1.807(3)	180.0(3)	180.0(2)
Cg1···Cg2	3.6313(12)	3.5891(12)	-3.5435(13)	0.552(3)	0.794(3)	120.6(4)	168.81(7)

[a] *Cg* are planes of the 3,4-dichlorophenyl moieties (**Figure 5**).
[b] R, distance between the centroid of one ring and the centroid of a π···π stacked ring;
[c] h_1 and h_2, distance between the centroid of one ring and the plane of a π···π stacked ring;
[d] r_1 and r_2, distance between the centroid of one ring and the projection of the centroid of a π···π stacked ring to the first plane (rings offset);
[e] φ, twist angle is defined as the angle between the plane of the anchoring ring and the plane containing six atoms of aromatic ring;
[f] θ, angle between planes of adjacent π···π stacked ring.

S4. Void channels in structures of *cis*-**A**·½(1,2-DBE) and *cis*-**A**·½C_6H_{14}

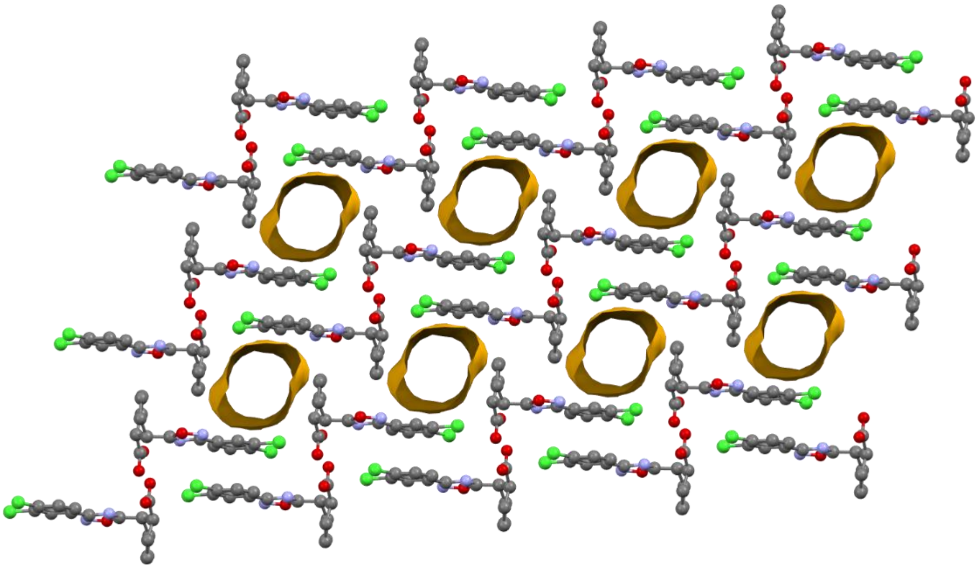

Figure S1. Partial representation (Mercury 4.3.1, ball and stick) of the crystal packing of *cis*-**A**·½(1,2-DBE) after orientations evidencing the cylindrical shape and the parallel arrangement of the channels (contact surfaces in ocher). A probe radius of 1.2 Å and an approximate grid spacing of 0.7 Å were used to generate channels. Solvent molecules in the voids and H-atoms are omitted for the sake of clarity. Color coding: grey, carbon; red, oxygen; blue, nitrogen; green, chlorine.

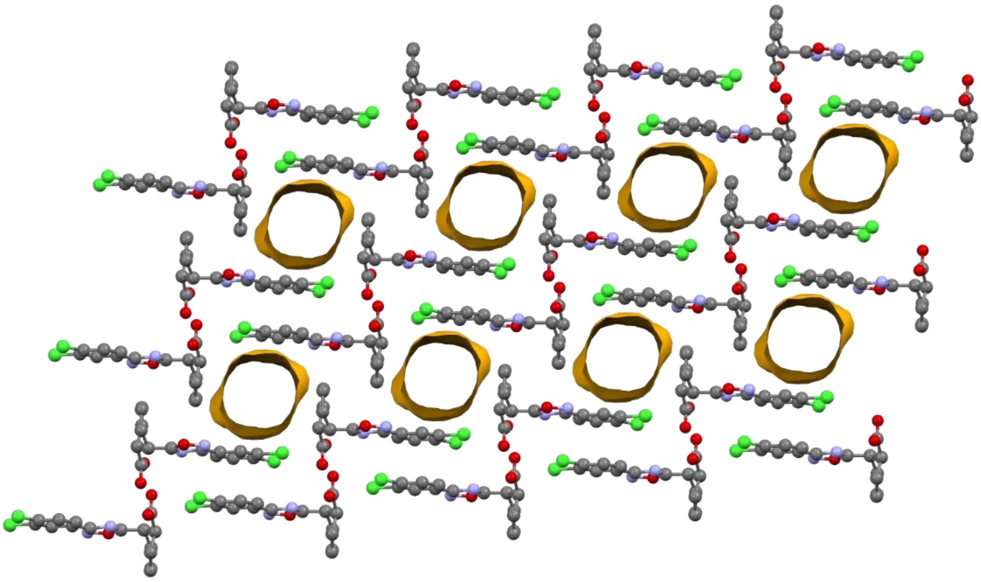

Figure S2. Partial representation (Mercury 4.3.1, ball and stick) of the crystal packing of *cis*-**A**·½C_6H_{14} after orientations evidencing the cylindrical shape and the parallel arrangement of the channels (contact surfaces in ocher). A probe radius of 1.2 Å and an approximate grid spacing of 0.7 Å were used to generate channels. Solvent molecules in the voids and H-atoms are omitted for the sake of clarity. Color coding: grey, carbon; red, oxygen; blue, nitrogen; green, chlorine.
